# Radiological and Biochemical Parameters in Assessing Acute Pancreatitis Severity: A Comprehensive Review

**DOI:** 10.7759/cureus.62288

**Published:** 2024-06-13

**Authors:** Shruthi Bikkumalla, Suresh R Chandak, Srinivasa Reddy, Poosarla Ram Sohan, Akansha Hatewar

**Affiliations:** 1 General Surgery, Jawaharlal Nehru Medical College, Datta Meghe Institute of Higher Education and Research, Wardha, IND

**Keywords:** multidimensional evaluation, prognostic significance, biochemical markers, radiological parameters, disease severity assessment, acute pancreatitis

## Abstract

Acute pancreatitis is a dynamic inflammatory condition of the pancreas with a spectrum ranging from mild to severe. Early and accurate assessment of disease severity is crucial for guiding clinical management and improving patient outcomes. This comprehensive review explores the role of radiological and biochemical parameters in assessing the severity of acute pancreatitis. Radiological imaging modalities, including computed tomography (CT), magnetic resonance imaging (MRI), and ultrasound (US), play a pivotal role in identifying key features, such as pancreatic necrosis and peripancreatic fluid collections, indicative of severe disease. Additionally, serum markers such as amylase, lipase, and C-reactive protein (CRP) provide valuable prognostic information and aid in risk stratification. Integrating radiological and biochemical parameters allows for a multidimensional evaluation of disease severity, enabling clinicians to make informed decisions regarding patient management. Early identification of severe cases facilitates timely interventions, including intensive care monitoring, nutritional support, and potential surgical interventions. Despite significant advancements in the field, there remain areas for further research, including the validation of emerging imaging techniques and biomarkers and the exploration of personalized management approaches. Addressing these research gaps can enhance our understanding of acute pancreatitis and ultimately improve patient care and outcomes.

## Introduction and background

Acute pancreatitis is a sudden inflammatory condition of the pancreas with varying severity. It can range from mild, self-limiting episodes to severe cases associated with significant morbidity and mortality. The condition often presents with abdominal pain, nausea, vomiting, and elevated pancreatic enzymes such as amylase and lipase [1]. Assessing the severity of acute pancreatitis is crucial for guiding appropriate management and predicting patient outcomes. Severe cases are associated with complications such as pancreatic necrosis, fluid collection, organ failure, and systemic inflammation. Early identification of severe pancreatitis allows timely interventions and improved patient care [2].

This comprehensive review explores the role of radiological and biochemical parameters in assessing the severity of acute pancreatitis. By examining the current evidence, we aim to provide insights into the utility of various imaging modalities and serum markers in predicting the severity of the disease. Additionally, we will discuss the challenges and limitations of existing approaches and explore potential avenues for future research in this field.

## Review

Pathophysiology of acute pancreatitis

Brief Overview of Pancreatic Anatomy and Function

The pancreas, a vital organ in the abdominal region behind the stomach, plays a pivotal role in digestion and blood sugar regulation. It is approximately six to 10 inches long and serves dual functions: exocrine, aiding digestion, and endocrine, regulating blood sugar levels. Its exocrine glands produce crucial enzymes, trypsin, chymotrypsin, amylase, and lipase, that facilitate the breakdown of proteins, carbohydrates, and fats. These enzymes are discharged into the duodenum through the pancreatic duct, contributing to the digestive process. Moreover, the pancreas harbors endocrine cells known as islets of Langerhans, which secrete hormones such as insulin, glucagon, and somatostatin directly into the bloodstream. These hormones regulate glucose levels and other hormonal functions [3-6]. The pancreas is pivotal in maintaining the body's overall health and well-being with its dual nature, serving both digestive and endocrine functions.

Etiology and Triggers of Acute Pancreatitis

**Figure 1:**
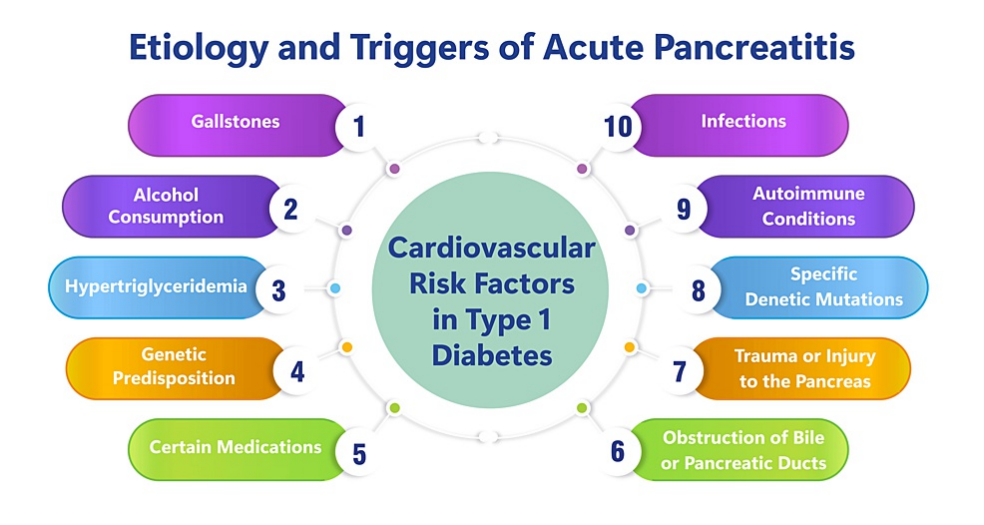
Eetiology and triggers of acute pancreatitis Image Credit: Dr Shruthi Bikkumalla

The etiology and triggers of acute pancreatitis encompass many factors capable of initiating inflammation within the pancreas. Among the most prevalent causes are gallstones and alcohol consumption, which collectively contribute to a substantial portion of cases [7,8]. Additionally, acute pancreatitis can be triggered by hypertriglyceridemia, post-endoscopic retrograde cholangiopancreatography (ERCP), genetic predisposition, certain medications, infections, autoimmune conditions, specific genetic mutations, pancreatic trauma or injury, elevated blood triglyceride or calcium levels, and obstruction of bile or pancreatic ducts [7]. This diverse range of etiological factors can precipitate the premature activation of pancreatic enzymes, leading to autodigestive injury to the gland and initiating the inflammatory cascade characteristic of acute pancreatitis [9]. Figure 1 shows the etiology and triggers of acute pancreatitis.

Pathophysiological Mechanisms Leading to Severity

The pathophysiological mechanisms leading to the severity of acute pancreatitis involve a complex interplay of various factors. Initially, the inappropriate activation of an inflammatory cascade is closely associated with the development of severe acute pancreatitis [10]. This inflammatory response, triggered by intra-acinar cellular trypsinogen activation and subsequent release of inflammatory mediators, significantly contributes to the disease progression [10]. Inflammatory markers and immune system activation emerge as pivotal areas for potential therapeutic interventions, underlining their critical role in comprehending and managing the severity of acute pancreatitis [11]. Moreover, the pathogenesis of severe acute pancreatitis is multifaceted, encompassing a three-phase continuum: local inflammation of the pancreas, a generalized inflammatory response, and the final stage of sepsis with multiple organ damage [11]. The disease process transitions from the premature activation of zymogen granules to SIRS, acute respiratory distress syndrome (ARDS), and multiorgan failure (MOF) [11]. This progression underscores the crucial involvement of inflammatory cytokines, proteolytic enzymes, and neutrophil-mediated reactions in driving the severity of acute pancreatitis.

Clinical presentation and diagnosis of acute pancreatitis

*Clinical Manifestation,* *Diagnostic Criteria, and Scoring Systems*

The symptoms and signs of acute pancreatitis typically manifest as sudden, severe upper abdominal pain that may radiate to the back, accompanied by nausea, vomiting, fever, rapid pulse, and a tender abdomen. Patients commonly report experiencing intense pain in the center of the upper abdomen, below the breastbone, which may escalate and extend to the back. Interestingly, leaning forward often relieves the pain, whereas lying down or walking may exacerbate it. Additional symptoms may include sweating, abdominal tenderness upon palpation, and worsening discomfort following meals. In severe cases, manifestations such as jaundice, hypovolemic shock, and organ failure may develop, necessitating prompt medical intervention [7,12,13]. The diagnosis of acute pancreatitis is typically confirmed by the presence of two of the following three features: 1) abdominal pain consistent with pancreatitis, 2) serum levels of amylase and lipase exceeding three times the upper limit of normal, and 3) characteristic findings observed on imaging studies such as CECT scans [14]. Various prognostic scoring systems have been developed to assess severity and predict outcomes, including Ranson's criteria, the Glasgow score, the MOSS score, the BISAP score, the SIRS score, and the APACHE II score [15]. However, the modified Marshall scoring system is often preferred due to its simplicity, universal applicability, and capacity to efficiently and objectively stratify disease severity [15]. Among individual biochemical markers, elevated C-reactive protein (CRP) levels have demonstrated good prognostic accuracy, with concentrations exceeding 150 mg/L, indicating a complicated course with 85% sensitivity [15]. Additionally, emerging biomarkers such as interleukin-6 (IL-6) and procalcitonin (PCT) have shown promise in early severity stratification of acute pancreatitis [16]. Radiological scoring systems, such as the Balthazar and modified CTSIs, offer further prognostic insights by evaluating both pancreatic and extrapancreatic findings [17].

Radiological parameters in assessing acute pancreatitis severity

Imaging Modalities and Key Radiological Findings Indicating Severity

Various imaging modalities play crucial roles in diagnosing and staging acute pancreatitis. Contrast-enhanced CT (CECT) is the imaging modality of choice, offering detailed insights into the pancreas and its surrounding structures [18,19]. Abdominal ultrasound (US) serves as a valuable tool, particularly in the initial phase of acute pancreatitis, aiding in assessing biliary stones as a potential cause and facilitating monitoring of pancreatic collections. However, its utility may be severely limited due to poor visibility [20]. While less frequently utilized than CT, magnetic resonance imaging (MRI) proves invaluable in specific scenarios. It is beneficial for evaluating patients with iodine allergies, characterizing collections, and assessing the pancreatic duct, especially in cases involving ductal disconnection [18,20]. Additionally, ERCP plays a significant role in identifying common bile duct stones in cases of acute biliary pancreatitis [20]. Crucial radiological indicators of acute pancreatitis severity encompass pancreatic enlargement, fat stranding, fluid collections, and pancreatic necrosis. These findings are pivotal markers when evaluating acute pancreatitis severity through imaging techniques such as computed tomography (CT) [17,19]. The CT severity index (CTSI) categorizes acute pancreatitis into five escalating levels of severity (A to E) based on these radiological features, furnishing essential insights for clinical decision-making and patient care [19]. Furthermore, the modified CT severity index (MCTSI) stands as another valuable tool, aiding in estimating parenchymal injury extent and enhancing the early prognostic capability of CT in acute pancreatitis. It facilitates the direct assessment of pancreatic damage [17]. These radiological parameters significantly sway forecasting acute pancreatitis severity and guiding appropriate treatment approaches.

Role of Imaging in Guiding Management Decisions

Imaging serves as a critical tool in directing management strategies for acute pancreatitis. CT imaging holds particular significance in evaluating the severity of the condition; assessing necrosis extent; identifying fluid collections, pseudocysts, and abscesses; and offering prognostic insights [21]. In diagnostic uncertainty surrounding acute pancreatitis, initiating an initial CT assessment is strongly advised, typically within 72-96 hours post-symptom onset. This aids in confirming severity, informing treatment decisions, and gauging the necessity for further interventions [21]. Furthermore, MRI equipped with fluid-sensitive sequences proves beneficial in evaluating debris presence within fluid collections and detecting ductal disruptions. This assists in managing acute pancreatitis cases complicated by these factors [21]. Beyond diagnosis, imaging modalities play a pivotal role in pinpointing the condition's underlying cause, such as distinguishing between biliary and non-biliary etiologies. This differentiation is crucial for tailoring treatment strategies effectively [21].

Biochemical parameters in assessing acute pancreatitis severity

Serum Markers

Several serum markers are utilized in diagnosing and assessing acute pancreatitis. Serum amylase, while commonly employed for diagnosis, may exhibit reduced sensitivity due to factors such as delayed presentation, hypertriglyceridemia, and chronic alcoholism [16,22]. Serum lipase is another vital marker for acute pancreatitis diagnosis, with elevated levels indicating disease severity. Notably, lipase levels may remain elevated longer than amylase after acute pancreatitis onset [22,23]. CRP emerges as a valuable marker for inflammation, capable of distinguishing between mild and severe acute pancreatitis. When used alongside PCT, CRP aids in early severity prediction [24,25]. PCT, known for its sensitivity, is a reliable predictor of severe acute pancreatitis, surpassing other tests in sensitivity and specificity. Its early predictive capability facilitates timely intervention [24,25]. Several other markers, including IL-6, polymorphonuclear elastase, serum amyloid A, and serum HGF, have been evaluated for their potential role in assessing acute pancreatitis severity [24,25]. These markers contribute to a comprehensive diagnostic and prognostic approach, enhancing the management of acute pancreatitis cases.

Prognostic Significance of Biochemical Markers

The literature extensively documents the prognostic significance of biochemical markers in evaluating the severity of acute pancreatitis. PMN-elastase and trypsinogen activation peptide (TAP) have emerged with heightened prognostic value in acute pancreatitis, facilitating prognosis and severity assessment [26]. Additionally, CRP and PCT have demonstrated potential in distinguishing between mild and severe acute pancreatitis cases, with PCT exhibiting superior sensitivity and specificity compared to alternative tests [24]. Moreover, studies underscore the importance of serum amylase and lipase levels in diagnosing and gauging acute pancreatitis' severity. Notably, serum lipase persists at elevated levels for a longer post-acute pancreatitis onset than amylase, rendering it a valuable marker for disease progression and severity assessment [24,27].

Utility in Predicting Complications and Guiding Treatment

The role of biochemical parameters in predicting complications and directing treatment in acute pancreatitis holds significant importance. Biochemical markers such as CRP and PCT have proven valuable in distinguishing between mild and severe cases of acute pancreatitis, facilitating early complication identification and guiding appropriate treatment approaches [28]. Furthermore, elevated amylase and lipase serum levels, well above normal ranges, serve as vital diagnostic indicators of acute pancreatitis severity. These markers aid in diagnosis and offer insights into disease progression and the likelihood of complications, thereby assisting in treatment decisions [29]. Biochemical parameters are critical in anticipating complications and shaping treatment strategies in acute pancreatitis. They furnish clinicians with valuable information for assessing condition severity, anticipating potential complications, and customizing treatment plans to enhance patient outcomes.

Combined approach: integrating radiological and biochemical parameters

Advantages of a Multifaceted Approach

A multifaceted marketing campaign employing various channels, including social media, advertisements, and direct mail, enhances reach and brand visibility, crucial as it typically requires an average of eight touchpoints to engage a customer [30]. This broader approach ensures increased visibility and engagement with potential customers. The adaptability and resilience inherent in a multifaceted marketing strategy shield a business from vulnerability if one channel becomes less effective. This flexibility allows for swift pivoting and experimentation with new tactics, ensuring continued effectiveness [30]. In addressing complex topics like mental health or chronic disease management, a multifaceted approach that considers multiple factors and outcomes yields a more comprehensive and nuanced understanding [31-33]. This approach is preferable over relying solely on a single metric or method. Evidence suggests that multifaceted interventions targeting multiple risk factors yield improved clinical outcomes, including reduced mortality and progression to end-stage kidney disease, compared to usual care [31,33]. Such interventions underscore the effectiveness of multifaceted strategies in improving outcomes. A multifaceted strategy is adept at catering to the diverse needs and preferences of various customer segments or patient populations, contrasting with a one-size-fits-all approach [34]. This versatility ensures that different needs are addressed effectively, increasing engagement and satisfaction.

Challenges and Limitations

The challenge of early and accurate severity assessment in acute pancreatitis is evident in the search results. While clinical scoring systems such as the Ranson score, APACHE II, and Glasgow score serve as useful tools for initial severity assessment, the effectiveness of radiographic scoring systems in predicting severe pancreatitis early in the disease process is limited. This limitation stems from the complexity and variability inherent in acute pancreatitis [35,36]. The issue of increasing antibiotic resistance further compounds the management of acute pancreatitis, as highlighted in the search results. The growing polyresistance of microorganisms to most antimicrobial chemotherapeutic agents exacerbate the situation. This poses significant challenges in managing infected pancreatic necrosis and septic complications [36].

A lack of consensus on optimal management approaches for acute pancreatitis is evident from the search results. Controversies and evolving guidelines persist in various aspects of acute pancreatitis management, including fluid resuscitation, nutrition, antibiotics and antifungals, analgesics, and complication interventions [37]. The importance of multidisciplinary and specialized care in managing acute pancreatitis is underscored in the search results. It is crucial to manage acute pancreatitis within a specialist tertiary referral center, with coordinated input from multiple subspecialty services [35]. Despite advancements in diagnostics and treatment, high mortality rates, especially in severe cases, remain a significant concern, as noted in the search results. Mortality rates continue to be elevated, particularly in cases involving pancreatic necrosis, infected necrosis, and fulminant acute pancreatitis [36].

Case Studies Demonstrating the Combined Use of Radiological and Biochemical Parameters

The integration of radiological imaging, particularly CT scans, with biochemical markers such as serum lipase, amylase, BUN, hematocrit, PCT, and LDH emerges as crucial for a comprehensive assessment of acute pancreatitis, as highlighted in the search results [38]. While CT scans offer valuable insights into local complications, necrosis, and severity, biochemical markers aid in diagnosis and initial severity evaluation. According to the search results, the timing of CT scans is paramount, with the most valuable window being 48-72 hours after symptom onset [38]. CT scans can more effectively differentiate between interstitial and necrotizing acute pancreatitis during this period. Early CT scans within the first 48 hours may have limited utility due to the dynamic nature of the disease. Despite their utility, radiological scoring systems such as the Balthazar CTSI have limitations in predicting severe pancreatitis early in the disease process, as indicated in the search results [39]. This limitation stems from the complexity and variability inherent in acute pancreatitis, leading clinicians to often prefer clinical scoring systems for initial severity assessment. The search results emphasize the necessity of a comprehensive approach integrating clinical, biochemical, and radiological parameters to accurately predict acute pancreatitis' severity [40]. No single parameter suffices; a holistic strategy encompassing these different aspects is essential for effective management.

Novel approaches and future directions

Emerging Radiological Techniques

Emerging radiological techniques in assessing acute pancreatitis encompass advanced MRI methods such as diffusion-weighted imaging (DWI) and MR elastography (MRE). DWI holds the potential to quantify signal changes indicative of underlying tissue abnormalities, thereby offering valuable insights into the pathophysiology of acute pancreatitis. Conversely, MRE with spin-echo echo-planar imaging provides a non-invasive means to evaluate tissue stiffness, aiding in the diagnosis and severity assessment of acute pancreatitis [41,42]. These cutting-edge imaging techniques extend beyond the traditional morphologic features assessed by CT and MRI, offering the prospect of unveiling imaging biomarkers that can refine classification and furnish crucial prognostic information in cases of acute pancreatitis. Moreover, incorporating radiomics and artificial neural networks (ANNs) augments the diagnostic prowess of these advanced imaging modalities, laying the groundwork for more precise and tailored management strategies for patients afflicted with acute pancreatitis [41].

Biomarkers Under Investigation

The biomarkers currently under investigation, as sourced, encompass a diverse range including IL-6, HADH, IL-1β, IL-8, IL-10, TNF-α, MCP-1, PCT, D-dimer, LY96, BCL2, IFNGR1, GSTM1, and lipase. These biomarkers are the subject of research aimed at uncovering their potential in predicting the severity of acute pancreatitis and distinguishing between acute and chronic pancreatitis. Notably, IL-6 has exhibited promise in prognosticating moderate severe or severe acute pancreatitis [43]. HADH, identified as a potential lipid metabolism-grounded biomarker, is scrutinized for its relevance to acute pancreatitis, particularly in patients with a BMI>30 [2]. Further investigations are underway on IL-1β, IL-8, IL-10, TNF-α, MCP-1, PCT, and D-dimer as potential predictive biomarkers for severe acute pancreatitis [44]. Similarly, LY96, BCL2, IFNGR1, and GSTM1 are subjects of study regarding their role as biomarkers in the emergence and progression of severe acute pancreatitis [45]. In addition, lipase levels are receiving attention as crucial biomarkers for acute pancreatitis and pancreatic cancer. Research efforts are focused on developing new fluorescent probes to enable sensitive detection [46].

Potential for Personalized Medicine in Acute Pancreatitis

The potential for personalized medicine in acute pancreatitis is substantial, with a burgeoning emphasis on precision medicine strategies that seek to customize treatment approaches to individual patients or specific patient subsets. This tailored approach entails pinpointing specific risk factors and predictors of post-acute pancreatitis diabetes mellitus (PPDM-A) through machine learning algorithms and analysis of clinical data. Advanced techniques like L1 regularized logistic regression models enable clinicians to diagnose PPDM-A early and offer personalized treatment recommendations based on individual patient attributes [47]. Moreover, the future trajectory of personalized medicine in acute pancreatitis involves the establishment of expert committees, such as the Pancreatitis Precision Medicine Interest Group, tasked with coordinating efforts across various centers to bolster the development of tailored treatment options. Precision medicine in acute pancreatitis aspires to alleviate the disease burden on patients and healthcare providers by prioritizing the unique needs of individual patients and specific subpopulations. This concerted effort holds the potential to yield more efficacious and precisely targeted therapies that enhance patient outcomes and overall quality of life [48].

## Conclusions

In conclusion, this review underscores the critical importance of accurately assessing the severity of acute pancreatitis and highlights the complementary roles of radiological and biochemical parameters in achieving this goal. By integrating imaging modalities such as CT, MRI, and US with serum markers like amylase, lipase, and CRP, clinicians can comprehensively evaluate disease severity, enabling timely interventions and improved patient outcomes. In clinical practice, this multidimensional approach holds significant implications, guiding treatment decisions, risk stratification, and patient care pathways. However, further research is warranted to refine existing scoring systems, explore novel imaging techniques and biomarkers, and elucidate the underlying pathophysiological mechanisms of acute pancreatitis. Through continued investigation and validation of emerging technologies, personalized management approaches can be optimized, ultimately enhancing our understanding of the disease and improving outcomes for patients afflicted with acute pancreatitis.
